# In-country availability of medical abortion medicines: a description of the framework and methodology of the WHO landscape assessments

**DOI:** 10.1186/s12978-022-01530-7

**Published:** 2023-01-24

**Authors:** Ulrika Rehnström Loi, Ndola Prata, Amy Grossman, Antonella Lavelanet, Natalie Williams, Bela Ganatra

**Affiliations:** 1 UNDP-UNFPA-UNICEF-WHO-World Bank Special Programme of Research, Development and Research Training in Human Reproduction (HRP), Department of Sexual and Reproductive Health and Research, World Health Organization, 20 Avenue Appia, 1211 Geneva, Switzerland; 2 Venture Strategies for Health & Development/OASIS, Berkeley, CA USA; 3 Bixby Center for Population, Health & Sustainability, University of California, Berkeley, USA

**Keywords:** Framework, Assessment, Medicines, Availability, Abortion, Mifepristone, Misoprostol, Combi-pack

## Abstract

**Background:**

Availability of quality-assured medical abortion medicines plays a crucial role in providing comprehensive abortion care. However, access to these medicines is still restricted for many abortion seekers. Increasing availability of affordable, quality-assured mifepristone and misoprostol is important to improve access to safe medical abortion services. Driven by the outcomes of a global consultation hosted by the World Health Organization and the Swedish International Development Cooperation Agency in 2018, we decided to holistically examine access to medical abortion medicines from supply to demand. The overarching principle of the national landscape assessments was to generate evidence to support policy dialog and policymaking that is contextual to the needs of the country. This paper aims to describe the framework and methodological approach used in the World Health Organization landscape assessments of medical abortion medicines at country-level.

**Methods:**

A country assessment protocol was developed to guide the methodology of the World Health Organization landscape assessments. The assessment protocol included adaptation of an existing availability framework, an online desk review and literature review for existing data available for the country of interest, country-level key informant interviews, and analysis of the data to identify barriers and opportunities to improve medical abortion availability.

**Conclusion:**

The availability framework and methodology will allow the identification of key barriers that limit readiness of medical abortion medicines, and the development of opportunities to overcome those barriers. The national landscape assessments will provide directions for future investments and offer guidance for policy and programming on medical abortion care.

## Plain Language summary

Increasing availability of affordable, safe, and effective medical abortion medicines is necessary to improve access to safe medical abortion services. Driven by the results of a meeting hosted by the World Health Organization and the Swedish International Development Cooperation Agency in 2018, we decided to examine access to medical abortion medicines from supply to demand in selected countries. The overarching goal of the national landscape assessments was to generate evidence to support advocacy efforts and policymaking that is appropriate to the needs of the country. This paper aims to describe the assessment protocol and the steps taken in the World Health Organization landscape assessments of medical abortion medicines at country-level. The assessment allows for a determination of the enabling environment surrounding the provision of medical abortion services. This in turn allows for the identification of key barriers that limit availability of abortion medicines, and the identification of opportunities to overcome those barriers. The availability framework includes both supply and demand sides of commodity availability, considering factors from product introduction to use. This approach can be used for future national assessments for any health commodity or service. The assessment findings will be informative to policymakers and programme managers in developing plans to safeguard availability of safe and effective medical abortion medicines.

## Background

Medical abortion (MA) with mifepristone and misoprostol is an important development that has contributed to the increased safety of abortion. MA medicines have successfully been registered in countries with a wide range of abortion laws and national policies that impact their availability, uptake and use in abortion care. While there is a significant pool of manufacturers contributing to wider availability, information on the quality of these products and their use in each country is limited and often not available. The lack of quality-assured, affordable active pharmaceutical ingredients (API) is also problematic. At present, there is only one mifepristone API and one misoprostol API prequalified [1].

Increasing the availability of affordable, quality-assured mifepristone and misoprostol is necessary to improve access to safe medical abortion services. Access to quality-assured medicines for medical abortion is still restricted for many women in need. One key barrier is affordability, limiting procurement and access to quality abortion medicines. The price of the medical abortion commodities remains a driving factor for procurers, which then influence choice, access, and availability. Most of the lowest priced medical abortion commodities available in the market are not quality-assured [2].

At a global consultation hosted by World Health Organization (WHO) and Swedish International Development Cooperation Agency (Sida) in January 2018, a group of experts identified critical gaps and developed an action plan to increase the global availability of low-cost, quality-assured, co-packaged mifepristone-misoprostol. Representatives from governments, donors, United Nations (UN) agencies, non-governmental organizations (NGOs), social marketing organizations, and manufacturers of medical abortion commodities agreed on the importance of the following actions to improve the global landscape of quality-assured co-packed medical abortion products.

1. Increase the number of quality-assured co-packed medical abortion products available for procurement and supply;
2. Increase awareness on quality of existing co-packed medical abortion products and support policy frameworks that allow for procurement of quality-assured co-packed medical abortion products;
3. Understanding the need for quality-assured co-packed medical abortion products is critical to identifying interventions to improve affordability and availability at regional and national levels.

Globally, more than 100 countries have registered misoprostol and/or mifepristone, with new country markets being assessed and product registered each year [3–5]. Sales of misoprostol and combi-packs have steadily increased [2, 6, 7]. Sales data may indicate increasing availability but lack description on how MA medicines are made available in both the public and private health sectors [8]. Country registration of a quality-assured product that can be used for MA—either misoprostol only or mifepristone + misoprostol separately or in a combination pack—does not necessarily mean that women will be able to access the product or MA services when needed. There are a host of other factors that influence the availability of MA services and commodities in countries [9]. These include service-delivery guidelines and other policies that influence where and when services can be provided and by whom, what products should be available, how products are registered, procured, and distributed, and how procurement is financed, provider knowledge about MA and the circumstances under which it can be offered, abortion stigma, and end-user knowledge of country abortion laws and services [10–12].

The agreed action points and documented lessons learned informed the development of a project to conduct national landscape assessments and develop country-specific recommendations to increase availability of affordable, quality-assured co-packaged mifepristone-misoprostol products in individual countries. Consequently, we developed a comprehensive assessment protocol including these factors. This paper aims to describe our methodological approach to assess the availability of MA medicines in countries by using an analytical and conceptual framework.

## Methods

The overarching principle of the landscape assessments was to generate evidence to support policy dialog and policymaking that is contextual to the needs of the country. The objectives of the national landscape assessments were to:

1. Conduct market assessments (a detailed and objective evaluation), including the regulatory landscape for introducing and/or scaling-up availability of co-packed MA products in selected countries;
2. Document specific needs, challenges and opportunities for expanding access to and availability of MA care in selected countries;
3. Develop feasible short-, medium-, and long-term plans for increasing access to and availability of co-packaged mifepristone–misoprostol which can include innovative approaches to increase availability and access to affordable, quality-assured co-packaged MA products and documentation of partners’ initial plans for introduction of a new, more affordable, quality assured MA commodity.

The development of the country selection criteria was based on discussions with WHO Regional technical staff. Factors such as opportunity to increase access to MA medicines, experience of conducting relevant work in the country and country need/request were considered during the selection process. In 2019, the first eight countries that were invited to participate in the Landscape Assessments were: Bangladesh, Liberia, Malawi, Nepal, Nigeria, Rwanda, Sierra Leone, and South Africa.

### Assessment protocol development

**Fig. 1:**
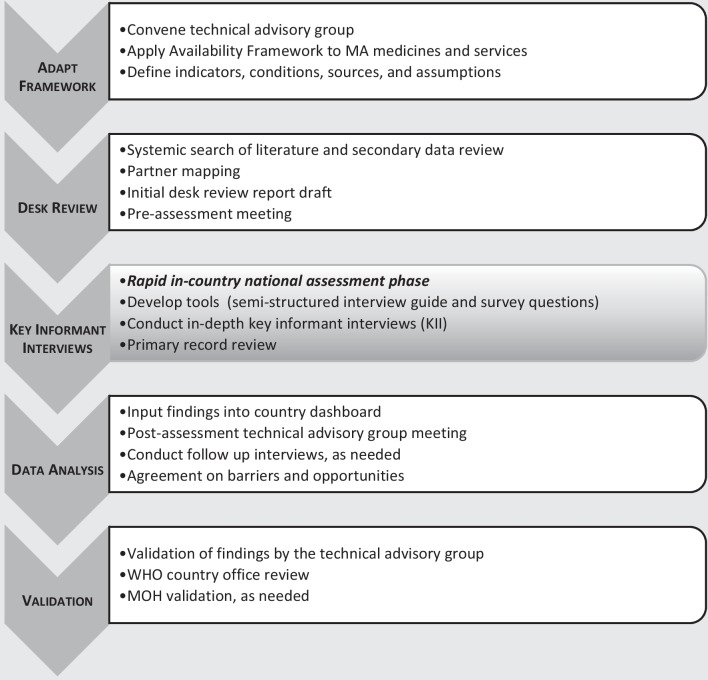
National assessment protocol

In order to set out the scenario, data and methods to be applied we developed a protocol to guide the country assessments on the availability of MA products (Fig. 1).

### Adaptation of the availability framework

**Fig. 2:**
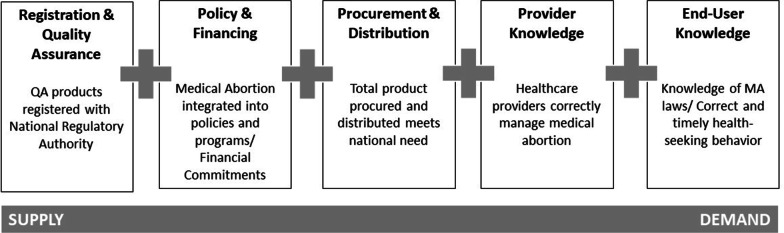
The availability framework applied to MA

Our methodology included an availability framework that consists of five areas or “pillars”; (1) registration and quality of assurance, (2) policy and financing, (3) procurement and distribution, (4) provider knowledge, and (5) end-user knowledge. The availability framework cover all aspects of availability and use of a commodity, from supply by the manufacturer to demand and use of the product or service by the end user. It was informed by two well-known analytical and conceptual frameworks for access to health commodities [13, 14] and was previously applied to assess misoprostol availability for postpartum haemorrhage in Tanzania and the availability of preeclampsia and eclampsia medicines and services in Ethiopia, Kenya and Nepal [15, 16]. A technical advisory group comprised of experts in pharmaceutical product development and regulatory affairs; public health program planning, monitoring and evaluation; and medicine, applied its collective experience and adapted the availability framework with the aim to conduct national assessments of the availability of medical abortion commodities in eight countries. We defined “availability” to mean that a woman can request and receive a high-quality and affordable MA product or service when and where she needs it. Figure 2 shows its application to MA medicines. The adaptation included revising the key indicators to align to MA medicines (Table 1).

**Table 1:** The expanded availability framework matrix for MA medicines

Pillar	Condition	Key indicator	Source/verification	Assumptions
Registration and quality assurance	Quality-assured (and other) products are registered and have correct MA indications on label; Products include: • Co-packaged mifepristone (1 × 200 mg) and misoprostol (4 × 200 mcg) • Misoprostol 200 mcg • Mifepristone 200 mg	Number of products registered Names of market authorization holder	List of registered products OR Certificate of registration/licensing	Product must be registered for use; Multiple registered products increase competition and coverage
		Medical indications Quality and accuracy of product label Convenient packaging	Product inserts/label (instructions for use) Packaging/ Presentation	Format and instructions for use influence actual usage
		Quality-assurance status—Stringent Regulatory Authorities, WHO Prequalification, UNFPA Expert Review Program	Product registration details or manufacturer licensing documents	Standards are sufficient to ensure quality; Manufacturers are complying with standards; Regulatory agency procedures outlined are sufficient to evaluate product quality
Policy and financing	Institutionalization of MA products and services in key national policy documents and guidelines Training and dissemination of policies and guidelines Commitment of public or private funds to procure products and provide services	Combination pack and/or individual drugs are: • On National EML and/or National Formulary (NF) • On National procurement list • Included in treatment guidelines	EML National Formulary; procurement list Treatment guidelines	Combination pack on EML or NF, procurement list drives procurement and availability
		Decreasing unsafe abortion is part of national strategy for MNH improvement	Maternal Health National Strategy Key informants	Key national policy documents establish the groundwork for programmatic plans
		Protocols for MA are in national medical/treatment guidelines and pre-service/in-service training curricula Mechanisms to disseminate information on MA MoH approved training plans and/or job aids	Standard/National Treatment Guidelines Emergency Obstetric Guidelines Training curricula	Appropriate and detailed guidelines are necessary to ensure safe use of MA Protocols influence practice Curricula are important for provider education
		Designation of funding for MA commodities in national budget/commodity budget	MoH annual budget/commodity budget	Government designates a budget line; Donor provides funding; Funds are allocated and disbursed when needed; Funds sufficient to cover product need, as defined by MoH strategy
Procurement and distribution	Total product procured and distributed by public and private sector meets national need Affordable pricing	Public sector/NGO/private procurement of QA-assured or other combi-pack • Volume procured and sold/distributed • Distribution channels • Cost—wholesale and retail • Forecasting tool used • Product in procurement tenders Sufficient budget allocated	Central Medical Stores records Distributor/importer procurement and sales records Pharmacies	There is public/private sector procurement Forecasting tool used and is accurate Products are purchased through tenders or special orders
		Stock-outs (public, private, NGO): • Reported/observed stockout per sector % of zonal stores that experienced a stockout of product at any point during the past year	Central/regional/district/medical stores data Medical Stores reports MoH programmatic plan CMS reports; Key informants	Public sector procurement is supposed to cover all public sector need Public facilities rely on consistent stock at zonal store level
Provider knowledge	Product is institutionalized in provider training curriculums Medical providers at appropriate levels of care know about product and can provide services	Knowledge of MA, products and policies Ability to provide MA services according to guidelines	Provider interviews; Service Provision Assessments	Drugs and equipment needed to provide service
		Safe abortion guidelines and job aids Providers trained using these guidelines	Provider interviews/facility visits/surveys	Guidelines include MA
End-user knowledge and behaviour	Women of reproductive age know about safe abortion/MA and national policy/law	% women of reproductive age that know about MA and safe abortion policies	Country-specific surveys and studies	If women know product’s uses, utilization will increase

The technical advisory group used a Logical Framework Matrix based upon several validated tools used to assess health commodity supply to adapt the availability framework to medical abortion and categorize key indicators [17, 18]. The expanded availability framework matrix applied to medical abortion includes a definition for each pillar, a rationale for inclusion, a list of key indicators, a means of verification, and assumptions where appropriate (Table 1). The development of indicators was informed by past programmatic efforts to introduce and scale-up misoprostol for obstetric indications and the medical and regulatory knowledge of the technical advisors. This adapted framework guided data collection and formed the basis of country-level dashboards, for each of the countries to be assessed.

For the Registration and Quality Assurance pillar, the primary indicator was the number of registered misoprostol, mifepristone, and/or combi-pack products approved by the National Regulatory Authority (NRA) in a given country. This included the names of market authorization holder (distributors/importer), the medical indication for which the products may be used and its quality-assurance status.

Within Policy and Financing, indicators included the country’s abortion law and its level of restriction, categorized as High (prohibited outright or only permitted to save the life of the pregnant woman), Medium (to preserve health and/or economic grounds) and Low (available by request) restriction countries, consistent with other recognized characterizations [19]. Indicators also included whether the combi-pack, mifepristone and/or misoprostol are (1) on National Essential Medicines List (NEML) and/or National Formulary; (2) on national procurement list; and (3) included in national standard treatment guidelines. The assumption was that inclusion in any one of these lists would drive procurement and product availability in the public healthcare system [20]. Additionally, we determined how these products are purchased, if at all, and any restrictions on their procurement or financing.

Within the Procurement and Distribution pillar, key indicators included public sector tenders for misoprostol, mifepristone, or combi-pack, their volumes and wholesale costs; public sector stock-out data; and private sector distribution points and retail costs. These indicators helped describe the coverage of product in a given country in both the public and private sectors.

The final two pillars are Provider Knowledge and End-user Knowledge. Key indicators of Provider Knowledge were the existence of national safe abortion guidelines, government-approved pre-service curricula and/or in-service training curricula, and published research on provider’s knowledge, attitudes and practices (KAP) related to abortion care in that country. The assumption was that national abortion policies would guide practice and enable correct use by clearly defining when, where, how and by whom abortion services, including medical methods, could be provided in the country. Key indicators of end-user knowledge were limited to country-level studies on women of reproductive age and their awareness of MA and safe abortion policies and laws. The assumption was that end-users’ knowledge of their right to abortion influences their ability to access and use abortion services, including MA. (Table 1.)

### Country-specific desk review

We conducted a systematic desk review for each country that included a targeted online search of indicators and information included in each of the framework pillars (Table 1). Primary sources of information included the country’s penal code and constitution as it related to termination of pregnancy; NEML, national formulary, and standard treatment guidelines. Secondary sources were reviewed to populate the framework and included government reports, national health survey census data, the WHO Global Abortion Policies Database, and peer-reviewed research and published program reports. Search terms on PubMed and Google Scholar databases included the country name and the key indicators being assessed (e.g. NEML, maternal health strategy, mifepristone, misoprostol, provider’s KAP in provision of abortion services) as well as general topics such as pharmacy access; MA; comprehensive abortion care (CAC); supply chain; regulatory affairs; and abortion-seekers. To identify possible MA products in-country we searched the NRA’s website of registered products by active product ingredient, if available. We crossed that information with several online databases to create a list of products to verify with the NRA [21, 22]. We searched NRAs’ websites for inclusion of processes related to good manufacturing process, safety, pharmacovigilance, quality assurance and dossier requirements.

Initial desk review reports were drafted and presented to the technical advisory group at a pre-assessment meeting. The presentation included a country overview, indicator results by pillar, and gaps in the literature and initial online scoping exercise. The group identified areas for additional inquiry and clarification, potential key informants to address gaps, and developed questions for key informant interviews (KIIs).

Concurrent with the desk review was a partner mapping exercise to identify NGOs, donors and government programs that focused on access to safe abortion, health systems strengthening, commodities and MA specifically. A database of NGO partners and potential key informants was collected and mapped to the specific areas of the framework they were likely to best address (e.g. Head of National Regulatory Authority for Registration & Quality Assurance; Ministry Director of Reproductive Health to Policy & Financing and Provider Training; President of National Obstetrician/Gynaecology Society to Provider Knowledge).

### Country-level key informant interviews

We developed semi-structured interview guides and survey questions to use during the national assessment. These tools were kept flexible to permit for customization for improved reflection on country context while retaining several core questions for cross-country comparisons. The tools were designed to address missing indicators; clarify and corroborate desk review findings; gain expert knowledge of the local situation, barriers and opportunities; and elicit new areas of inquiry.

Initial meetings were held with Ministry of Health and WHO Country Office staff to validate the initial interview list and plan for the KIIs. Ethics approval was generally not applicable as these country assessments were led by the ministries of health as programme assessments and not conducted as research activities. In countries where required, ethics approval was obtained. The information collected during the desk review is publicly available data and the key informants all participated within their official capacity and were selected by the ministries of health.

Snowball sampling among in-country partners and initial interviewees refined and expanded the interview list. Verbal informed consent to participate in the assessment was obtained from all participants. We conducted interviews either in-person or virtually and when unavoidable, emailed a survey for the informant to complete. The number of KII varied per country, but we conducted a minimum of 20 KIIs in each country with representatives from government, United Nations (UN) agencies, healthcare providers, professional medical and midwifery/nursing societies, international and local NGOs, and local wholesalers and pharmacies. We tailored interview guides to the interviewees’ area of expertise and focused questions related to framework key indicators, verifying information gathered in the desk review, gaining their expert knowledge of perceived barriers to availability, and elicit country-specific insights on abortion care. We conducted a record review of key documents in-country (e.g. NEMLs, training curricula, and standard treatment guidelines) that could not be found during the desk review, or included more recent versions than those available online. Wherever possible, document review of the regulatory certificate was obtained from the NRA or market authorization holder. The manufacturer’s name was then cross-referenced with the WHO Prequalified Lists.

### Data management and descriptive analysis

We organized national data in a country-specific dashboard of findings that focused on the primary indicators for each pillar. To maintain the confidentiality of interviewees, the database was password protected. The technical advisory group debriefed for a post-assessment meeting to determine strengths (where key indicators were met) and gaps (where indicators were missing or incomplete) for each country. We followed up with key informants to address gaps or points of clarification. We elicited themes from KIIs and organized and described them by framework pillar to define country-specific barriers to the availability of MA medicines and services and identified opportunities to improve availability. Data from KIIs and the desk review, as well as policies and other critical documents such as clinical guidelines and other documents were triangulated, verified, assessed for consistency and discussed amongst the technical advisory group. Remaining clarifications were identified and key stakeholders in the country were identified to resolve inconsistencies and gaps in information.

### Validation

With inputs from the technical advisory group, an initial report was developed to address the national landscape assessment objectives. Data from KIIs and the entire desk review was triangulated, assessed for consistency and discussed in a round table. Remaining clarifications were identified and key stakeholders in the country were identified to resolve inconsistencies and gaps in information. The draft report was circulated to the relevant WHO offices and Ministries of Health for validation.

## Discussion

This paper presents a methodological approach used in the World Health Organization landscape assessment of medical abortion medicines at country-level. To our knowledge a number of frameworks exist to assess availability of health commodities [13, 14, 23], but none have been applied to misoprostol, mifepristone, or the combi-pack. A number of studies document factors that affect access to abortion services, including restrictive abortion laws, stigma, poor quality or lack of medicines, lack of accurate information among providers and women, provider reluctance or conscientious objection, stigma, prescription requirements, and pricing [9, 24–26]. However, existing data captures information about each of these factors alone, but not across one country-context making it difficult for use by governments and program planners in-country.

## Strengths and limitations

A strength of this methodological approach to assess the availability of MA medicines in countries is that it prioritizes key indicators from product introduction to its use to assess availability at the country-level in a relatively short period of time (weeks to months, instead of months to years). Moreover, this holistic approach gathers and collates information across the framework pillars and is useful to governments and program partners who may only be active in one component area of availability. For instance, procurement officers and central medical stores may be unaware of new training sessions or community sensitization efforts conducted or planned in one area of the country, and mis-quantify the need or allocation of product to facilities in these catchment areas, leading to stock outs. Importantly, the availability framework couples the commodity supply-side components with provider and end-user knowledge. These latter two components ensure acceptance, demand for and adoption of a health commodity at the facility and community-levels. They are often omitted in discussions of market availability studies which heavily focus on commodities and the regulatory environment. Understanding demand for services is important to anticipate the potential market size and whether enough demand exists for multiple suppliers in a given market. Stand-alone KAP surveys of physicians and non-physician cadres and some country-level survey data begin to address service delivery factors that influence providers’ willingness to provide abortion care, but then rarely connect those findings to that of the supply and upstream availability dynamics [24, 26–28].

There are several limitations of our national assessment protocol. To be more representative, an assessment could include provider and retail surveys, however that is time-consuming and costly. We rely instead on the existing literature and KII. Depending upon the commodity of interest, there may be a paucity of literature on provider and end-user KAP related to its use. We use proxy measures in our methodology (Table 1). We assume that the existence of national service delivery guidelines and training curricula would guide practice and enable correct use and that such policies had been shared and read by providers, which may not always be the case in practice. KII with deans of teaching hospitals and/or heads of professional medical societies and clinicians attempted to answer some questions related to provider awareness and training (e.g. existence of curricula or specific in-service trainings conducted, dissemination of protocols). We acknowledge that assumptions about end-user knowledge of their legal right to abortion and ability to access services is simplistic; end-user access to services also relies upon other important potential barriers such as knowledge of where to access services and medicines, distance to points of consumption, transportation, and costs, which was beyond the scope of this assessment and may or may not be documented elsewhere. Additional research may be needed to further refine and evaluate the framework methodology in this regard.

## Conclusion

This assessment approach may be considered a protocol that can be applied for future national assessments for any health commodity or service. The availability framework includes both supply and demand sides of commodity availability, taking into account the interplay of factors from product introduction to use. The national landscape assessments would serve as a resource for countries to develop actionable strategies to ensure availability of quality-assured medical abortion medicines.

## Data Availability

The datasets used and/or analysed during the current study are available from the corresponding author on reasonable request.

## Abbreviations

API: Active pharmaceutical ingredient
CAC: Comprehensive abortion care
KAP: Knowledge, attitudes and practices
KII: Key informant interview
MA: Medical abortion
NEML: National Essential Medicines List
NRA: National regulatory authority
VSHD: Venture Strategies for Health & Development
WHO: World Health Organization
